# The Role of Collagens in Atopic Dermatitis

**DOI:** 10.3390/ijms25147647

**Published:** 2024-07-12

**Authors:** Krzysztof Szalus, Magdalena Trzeciak

**Affiliations:** Department of Dermatology, Venereology and Allergology, Faculty of Medicine, Medical University of Gdansk, 80-214 Gdansk, Poland

**Keywords:** atopic dermatitis, collagens, extracellular matrix, pathogenesis

## Abstract

Atopic dermatitis (AD) is a chronic inflammatory skin disease affecting both children and adults. The clinical picture of AD manifests in typical skin lesions, such as localized eczema and dry skin, with dominant, persistent itching that leads to sleep disturbances. The pathophysiology of AD has been extensively investigated with respect to epigenetic and genetic factors, skin barrier defects, as well as immunological and microbial disorders. However, to date, the involvement of extracellular matrix (ECM) elements has received limited attention. Collagen, a major component of the ECM, may serve as a therapeutic target for the future treatment of AD. This paper summarizes the role of collagens, which are the most abundant components of the extracellular matrix in AD.

## 1. Introduction

Atopic dermatitis (AD), also known as atopic eczema, is a chronic inflammatory skin disease characterized by flares. It affects both children, with a prevalence of up to 20%, and adults, with a prevalence of 4.9% [1]. This critically decreases the quality of life of patients and their families. The pathogenesis of AD is complex. Genetic and environmental factors lead to both epidermal and immune system disorders [2]. Genetic changes, including loss of filaggrin (FLG) function mutations, which occur in up to 30% of the AD population, are associated with early onset and severe AD courses [3]. However, it is worth mentioning that the FLG mutations differ across ethnicities.

The skin barrier plays a key role in protecting the body against external factors such as pathogens and allergens. A faulty barrier makes the skin susceptible to dryness, environmental irritants, and allergens, leading to inflammation. It is believed that dermal defects may be partially caused by reduced levels of ceramides or sphingolipids in the stratum corneum, which prevent transepidermal water loss (TEWL), FLG mutations, a lack of natural moisturizing factor (NMF), elevated pH of the skin, proteinase hyperactivity, microbial dysbiosis with *Staphylococcus aureus* domination, and defects in natural immunological defense associated with a lack of antimicrobial peptides. All these factors contribute to the penetration of irritating substances and allergens into the skin, ultimately reaching targets that trigger an immunological response. The response appears due to the excessive expression of Th2 lymphocytes; an increase in the production of pro-inflammatory cytokines IL-4, IL-5, IL-13, and IL-31; and increased expression of Th1 lymphocytes and IFN-gamma with IL-12 in chronic lesions [4]. Mechanical damage to the skin, such as scratching, also stimulates keratinocytes to release inflammatory cytokines such as alarmins, IL-33, IL-25, and thymic stromal lymphopoietin (TSLP) [5].

The development of AD is intricate and involves factors such as a disruption in the skin barrier, disturbances in immune mechanisms, and alterations in the skin microflora. In addition, numerous environmental and personal factors play a role in shaping the course of AD.

### 1.1. Skin Barrier Defect

Filaggrin (FLG) stands out as the foremost keratinized envelope protein implicated in the pathogenesis of AD. The absence of FLG leads to dry skin, and reduced FLG expression arises from gene mutations and chronic inflammation of atopic skin [6]. Alongside the altered expression of cornified envelope (CE) proteins such as FLG, lorikrin (LOR), cornulin (CRNN), repetin (RPTN), and small proline-rich proteins (SPRRs) [7], the stratum corneum suffers from deprivation of intracellular lipids such as ceramides [8]. Notably, skin barrier defects manifest in both normal and unaffected skin and affected areas in AD patients [9]. Epidermal barrier dysfunction results in increased permeability, reduced epidermal cohesion and integrity, increased transepidermal water loss (TEWL), skin dryness, and the occurrence of skin cracks and ruptures [10]. Such damage to the epidermal barrier facilitates allergen penetration, leading to epidermal sensitization and the onset of allergies [11].

### 1.2. Immunologic Disorders

Immunological dysfunctions in AD encompass both innate and adaptive immune responses. The primary pathological pathway of AD hinges on the activation of the Th2 lymphocyte axis in the inflammatory process [12]. Various triggers, such as mechanical injuries, allergens, and invasive microbiota, can provoke and expedite immune mechanisms in the skin, leading to a rapid increase in IL-25 and IL-33 expression in the skin’s innate immune system. This, in turn, further stimulates the Th2 lymphocyte response cascade. Th2 lymphocytes also play a pivotal role in producing IL-31, dubbed the “pruritus cytokine,” which is abundant in acute skin lesions, contributing to the itch–scratch cycle alongside other mediators such as histamine, tryptase, and neuropeptides. Although the acute phase of AD is traditionally thought to be strongly influenced by Th2 and Th22 lymphocytes, recent investigations have highlighted the significant impact of Th17 lymphocytes, IL-17, and IL-23 on modulating the pathology of the acute phase of AD. Although Th17 lymphocytes are primarily recognized as fundamental mediators of psoriasis through IL-17 production, in AD, IL-17 sustains the inflammatory process and serves as a chemokine for neutrophils and T lymphocytes [12]. Consequently, the acute phase of AD is predominantly driven by the activation of Th2 and Th22 lymphocytes, whereas chronic lesions demonstrate the influence of Th1 lymphocyte activity. Activation of the Th1 lymphocyte pathway leads to the upregulation of interferon (IFN) gamma and IL-12, promoting the chronic phase of inflammation and keratinocyte apoptosis [12]. Furthermore, disparities exist in the immunological implications between pediatric and adult skin. While both exhibit significant activation of the Th2 axis, early-onset AD in the pediatric population is notably associated with IL-17-related inflammation. Conversely, adults show an increase in Th22 lymphocyte polarization, likely correlated with prolonged immune stimulation over time [13]. This suggests that AD may encompass different subtypes based on molecular immunotypes/genotypes [14].

Different ethnic groups present variations in the immune response to atopic dermatitis (AD). This heterogeneity of AD presentation is likely due to variations in the immune response. Endotyping AD according to different ethnic groups is crucial for establishing disease biomarkers and developing precise therapeutic approaches. The Asian type of atopic dermatitis shows increased expression of TH17. These results contrast with the data on the skin of European subjects, who did not show activation of the TH17 axis [15]. This results in different IL-19 levels, which were significantly higher in AD lesions in Asian patients than in EA patients, since they were induced by IL-4, IL-13, as well as IL-17 and potentiated the effects of IL-17 on keratinocytes [15,16]. In addition, Chinese patients with AD exhibit a unique clinical phenotype compared to European patients [17]. Among the Chinese population, it has been shown that there is a strong activation of TH2 cytokines and chemokines (IL-4, IL-13, IL-5, IL-10, IL-31, and CCL13/17/18/22/26) [18].

### 1.3. Microbiota in AD

AD dysbiosis and reduced diversity of physiological microflora are well-documented conditions in AD. Research has shown a correlation between the dominance of highly virulent *Staphylococcus aureus* strains during atopic dermatitis (AD) flares and the increased severity of the condition [19]. In healthy, non-lesional skin, a balance between commensal bacteria and *S. aureus* colonization exists, contributing to the maintenance of skin defense mechanisms and protection against invasive microbiota [20]. However, in AD, the lack of antimicrobial peptides and innate immunity disorders, including Toll-like receptor dysfunction, create an environment favorable for the proliferation of virulent *S. aureus* strains [21].

*S. aureus* employs various mechanisms to compromise the skin barrier, disrupting the integrity of the epidermis and dissolving the stratum corneum [22]. Its virulence mechanisms involve the action of enterotoxins, alpha-delta toxins, and proteases, which, through interactions with Th-lymphocytes, mast cells, dendritic cells (DCs), and IL-31, intensify sensations of itching [23].

### 1.4. The Role of Collagens

**Figure 1:**
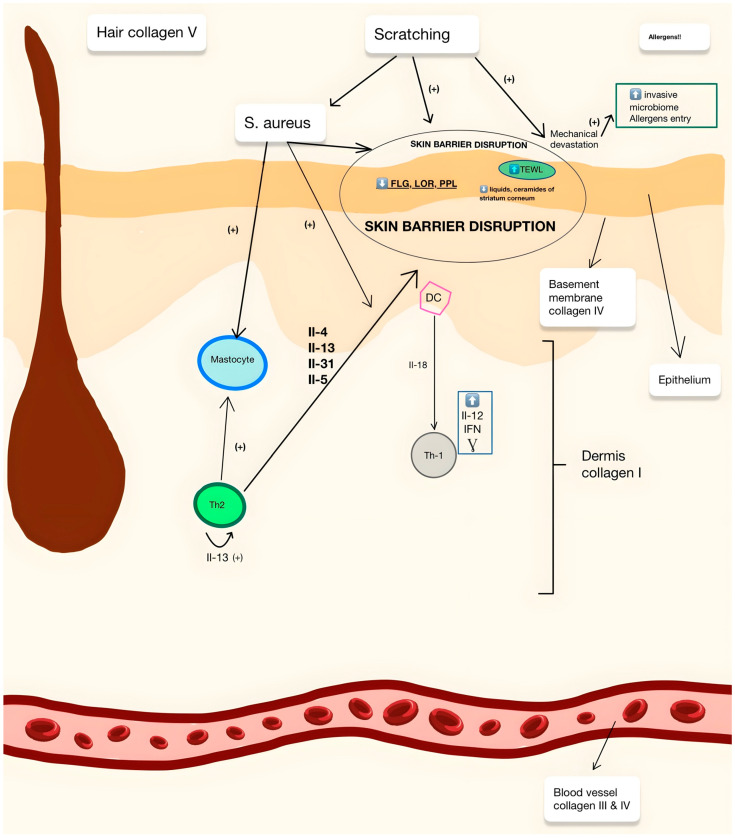
The pathogenesis of AD and the distribution of major collagen deposits in the skin (Th2—T2 helper lymphocyte; Th1—T1 helper lymphocyte; IFN—interferon; DC—dendritic cell).

Collagens are an integral component of skin; see Figure 1. Lately, extracellular matrix components, collagens in particular, have attracted the attention of scientists and physicians to state their role in AD. The ECM is a highly responsive and diverse structure that plays a key role in regulating tissue development, cell adhesion, and intercellular communication. Additionally, it acts as a store for growth factors and cytokines. This process plays a critical role in development, wound healing, and maintaining proper organ homeostasis [24]. Additionally, collagens in the ECM may play an important role in the skin’s immune response by influencing the migration of Langerhans cells and epidermal T lymphocytes that present antigens [25]. Fibroblasts and keratinocytes are the main drivers of ECM remodeling in adult skin because they produce many ECM proteins and secrete enzymes that degrade the matrix [26]. Fibroblasts also synthesize various matrix-degrading enzymes, such as matrix metalloproteases (MMPs), tissue inhibitors of MMPs (TIMPs), serine proteases (e.g., plasmins), granzymes, elastases, and cathepsins, which hydrolyze collagen, elastin, glycoproteins, and PG/GAG. Different types of fibroblasts have unique matrix secretion profiles. For example, fibroblasts expressing DPP4 produce higher amounts of type I collagen and fibronectin compared to fibroblasts without DPP4 expression. Additionally, immune cells secreting numerous cytokines and growth factors such as IL-1, IL-4, IL-6, IL-13, TGF-β, and TNF can stimulate ECM synthesis or degradation [27]. MMP-dependent ECM degradation induced by immune cells also contributes to the progression of AD. Therefore, patients with atopic dermatitis have elevated serum concentrations of MMP-8 and MMP-9. Moreover, increased macrophage infiltration in the chronic phase of the disease may disrupt ECM homeostasis through increased MMP activity [28]. Chronic atopic dermatitis is characterized by skin remodeling leading to fibrosis, a consequence of aberrant tissue repair triggered by various factors, including allergic reactions. Chronic inflammation, characterized by increased expression of various pro-inflammatory cytokines and chemokines, leads to the activation of fibroblasts and, as a result, the overproduction of ECM [29].

## 2. Collagens

Collagen is a protein with a significant molecular weight that constitutes the foundation of the skin, giving it structure and resistance to stress. Collagens are the main components of the extracellular matrix (ECM), which constitutes the structural support of the skin, contributing to its tension, elasticity, and resistance to damage [30]. Collagen molecules are evolutionarily conserved in multicellular organisms and contain numerous glycine (Gly)-X-Y repeat sequences. In these sequences, both X and Y are often proline and hydroxyproline. Collagens are primarily synthesized and released by fibroblasts; however, studies have shown that other cells, including basal keratinocytes, can also express some collagens, such as type VII collagen [31]. Fibrous collagens I, III, and V are the major types found in the dermal interstitial matrix. After birth, type I collagen becomes the predominant form in the dermis of adult skin.

Type I collagen constitutes approximately 80–90% of the total collagen content in the skin, while type III collagen accounts for the remaining 10–20%. These collagens play a key role in maintaining the structure and elasticity of the skin. They are part of the skin’s extracellular matrix, providing structural support, tension, and resistance to damage [32].

Type IV collagen is the main structural component of the basement membrane. It differs structurally from type I collagen in that it has intramolecular discontinuities of successive Gly-X-Y motifs, which allows for greater flexibility and the formation of a mesh-like network rather than the stiff, fibrous structure characteristic of interstitial ECM.

Type VI collagen is filamentous and forms beaded microfibrils that connect type IV collagen with type I and III collagen in the papillary dermis. Type VI collagen plays a key role in maintaining skin tissue homeostasis [33].

**Figure 2:**
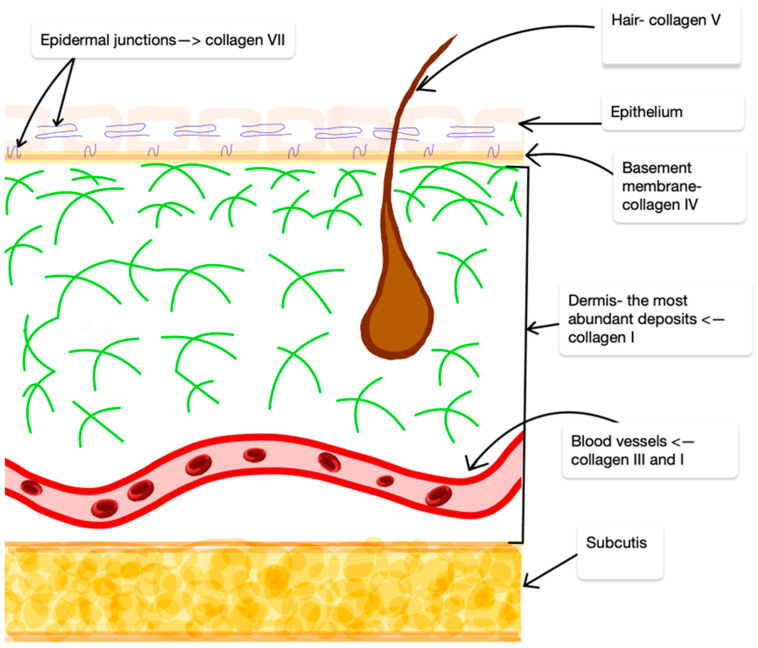
Distribution of individual collagens in the skin structure in relation to individual layers of the skin.

Type VII collagen is composed of anchoring fibrils located in the interphase between the basement membrane and the dermis of the papillary layer and is important for stabilizing the dermal–epidermal junction. As such, type VII collagen can maintain the integrity of the basal membrane interstitial matrix by interacting with type IV collagen [34] (Figure 2).

Inflammatory skin diseases can change the composition and structure of the extracellular matrix. In healthy skin, type IV collagen forms a network with interspersed laminin to form the basement membrane. This provides attachment points for primary epidermal keratinocytes and creates a protective layer that controls the navigation of leukocytes through customized gap sizes and limits the entry of harmful agents.

Despite the multifactorial etiology of AD, some evidence suggests that extracellular matrix components, particularly collagens, may play a role in the disease [35]. Both type I and type III collagens are abundant in the skin and play important roles in its structure. Studies have shown that patients suffering from atopic dermatitis often exhibit changes in collagen expression and degradation, which may impact the integrity of the skin barrier. Moreover, changes in the collagen microenvironment may affect inflammation in the skin and worsen the symptoms of atopic dermatitis [36]. Furthermore, the role of collagens in wound healing and skin repair is an intrinsically relevant research topic in the context of atopic dermatitis, given the skin damage caused by the scratching and itching cycle [37].

Genetic variations within collagen synthesis genes have been shown to impact the structure and function of connective tissue, as highlighted by several studies [38]. These differences translate into increased susceptibility to mechanical stress and compromised epidermal integrity and ultimately contribute to the aging process. Chronic AD skin with lichenification likely exhibits these consequences to a greater extent. Beyond structural support, the collagen proteins within the ECM actively influence the skin’s immune response. This occurs through their impact on the migration of Langerhans cells and T-antigen-presenting epidermal cells [39].

## 3. Collagen in AD

Studies show that patients suffering from atopic dermatitis often show reduced expression of type I and III collagens in the skin compared to healthy people. These changes can affect the integrity of the skin barrier and increase skin permeability, leading to increased inflammation and itching. Furthermore, atopic dermatitis, a T helper cell type 2 (Th2)-mediated inflammatory skin disease, exhibits a reduction in the abundance of type IV collagen and fibronectin, leading to a diminished basement membrane thickness. Increased levels of MMPs are also characteristic of atopic dermatitis.

**Figure 3:**
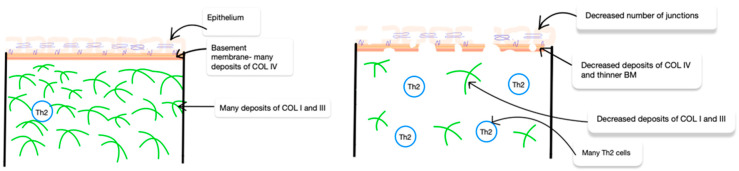
A comparison of general ECM in the healthy skin and in the AD skin with individual elements of a simple scheme of AD pathogenesis.

These enzymes can lead to the degradation of collagen in the skin, which weakens its structure and elasticity [40] (Figure 3).

Recent research in 2023 has explored the influence of ECM-regulating genes on AD pathogenesis, unveiling specific genetic variants in COL3A1 and COL6A5 associated with distinct clinical courses of AD. Studies have shown that polymorphisms in the COL3A1/rs1800255 and Col6A5/29rs12488457 genes may be associated with the clinical course of atopic dermatitis (AD), potentially influencing both disease severity measured by the SCORAD scale and the intensity of itching. Notably, the AA genotype in COL3A1/rs1800255 correlates with a milder course, while the GG genotype suggests a more severe AD progression, marked by intense pruritus. Similarly, the Col6A5/29rs12488457 gene shows lower mean SCORAD scores in patients with the AA genotype than in those with the AC genotype. These findings suggest that polymorphisms within these collagen-encoding genes could serve as population-specific biomarkers for AD, aiding in both disease predisposition assessment and personalized treatment strategies [41]. Another clinical study delved into the field of type 2 inflammation, investigating the effects of collagen tripeptide (CTP) characterized by a high content of Gly-X-Y tripeptides. The study demonstrated a significant reduction in inflammatory cytokine levels in keratinocytes after CTP treatment, highlighting its potential to inhibit inflammatory processes in AD-like conditions. Moreover, CTP treatment led to a decrease in rash area, atopic dermatitis scores (SCORAD), and transepidermal water loss (TEWL). These promising results indicate that CTP may serve as a therapeutic strategy for atopic dermatitis and other skin inflammations associated with type 2 inflammation [42].

**Figure 4:**
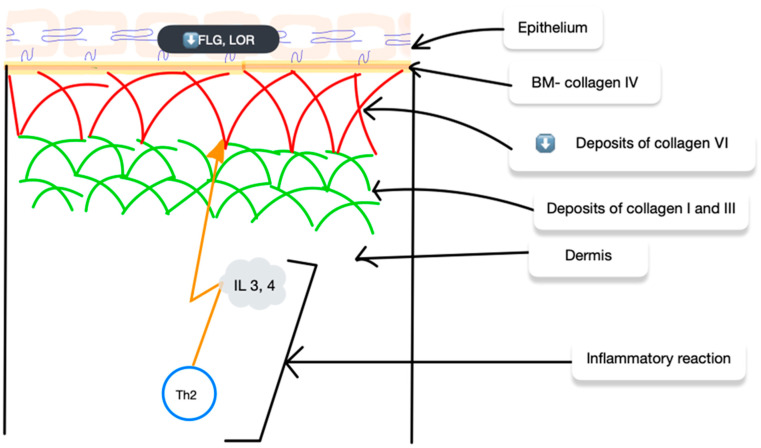
Immunological mechanisms that influence changes in collagen deposits and, further, the weakened protective properties of the skin in AD and its general condition.

Given these promising results, the research spotlight has turned to collagen type VI (COL6), a protein that has been relatively unexplored in the field of dermatology. Specific polymorphisms in the gene encoding the α6 chain of collagen VI (COL6A6) were associated with early AD in Koreans. It is worth mentioning that a case–control study identified three variants in the COL6A6 gene as being more common in the AD patients than the controls. These studies also revealed reduced COL6A6 expression in the epidermis of the AD patients. Experiments exposing keratinocytes to IL-4 and IL-13 showed that these cytokines inhibit COL6A6 expression, subsequently affecting other key skin barrier proteins, such as FLG, LOR, and CASP14, consequently weakening the skin’s protective barriers [43]. Although promising, these results necessitate further investigation to elucidate the mechanisms underlying these associations. In AD patients with increased IL-4 and IL-13 expression, decreased COL6A6 levels in the epidermis correlate with reduced expression of key skin barrier proteins, potentially influencing the overall AD condition [44] (Figure 4).

In atopic dermatitis, elevated levels of IL-4 and IL-13 coincide with reduced expression of the crucial skin barrier protein COL6A6 in the epidermis. This decrease in COL6A6, potentially mediated by type VI collagen, is associated with a decline in the production of other key skin barrier proteins.

A recent 2023 cohort study investigated the role of collagen type VI (C6A6) in atopic dermatitis. The study demonstrated increased C6A6 levels in patients with AD compared to healthy donors. Moreover, C6A6 levels correlated with AD severity and decreased after immunomodulatory treatment, suggesting its potential as a biomarker for assessing disease severity and monitoring treatment response [33,45].

In another study, which focused on eczema herpeticum (EH), genetic risk factors associated with EH occurrence were explored. The analysis identified a heterozygous SNP (rs2973744) in the gene encoding collagen type XXIII alpha 1 (COL23A1), significantly associated with EH in patients with AD. This SNP may influence COL23A1’s ability to better defend against HSV-1 infection, affecting its expression, processing, or functioning. The presence of a soluble form of COL23A1 may limit the infection of keratinocytes with the HSV-1 herpes virus, while blocking the degradation process and release of COL23A1 into the extracellular matrix (ECM) increases susceptibility to infection by HSV-1 [46]. Another study corroborated these findings, showing that M2 macrophages with the rs2973744 COL23A1 SNP were more susceptible to HSV-1 infection compared to macrophages from healthy individuals. Therefore, M2 macrophages with the rs2973744 COL23A1 SNP were shown to be more susceptible to HSV-1 infection compared to macrophages from healthy individuals. M2 macrophages from healthy donors that expressed high levels of COL23A1 were less efficiently infected with HSV-1 [47,48].

## 4. Collagens and Possible AD Treatment

Understanding the role of collagens in atopic dermatitis may lead to the development of new therapeutic strategies. One potential approach is to stimulate collagen synthesis or reduce collagen degradation to restore skin barrier integrity in patients with atopic dermatitis.

The available literature also includes recent reports on attempts at therapeutic use of collagen preparations in the form of emollients delivered via nanoliposomes. One such study investigated H.ECMTM nanoliposomes, which contained soluble proteoglycan, hydrolyzed collagen, and hyaluronic acid. In a study involving 25 participants with mild AD or dry skin, daily application of H.ECMTM nanoliposomes for four weeks showed significant improvements in a reduction in itching, TEWL, and skin hydration. Immunofluorescence studies indicated increased expression of filaggrin and loricrin, while TNF-α expression decreased, suggesting an anti-inflammatory effect. In vitro and ex vivo studies confirmed the restoration of skin barrier function. Although promising, it is important to note the study’s limited sample size [45].

## 5. Conclusions

Atopic dermatitis remains an incompletely understood disease, prompting ongoing research efforts. It has also been shown that patients suffering from atopic dermatitis, an inflammatory disease based on a large share of the response mediated by type 2 T helper lymphocytes, often present reduced expression of type I and III collagen as well as a limited amount of type IV collagen and fibronectin in their skin compared to healthy people. This results in a thinner basement membrane and, as a consequence, may affect the integrity of the skin barrier and increase skin permeability, leading to increased inflammation and itching. As far as collagen disorders are concerned, an open question remains: are the causes a result of chronic inflammation and/or of the scratching/pruritus circle?

Collagens are an integral part of the skin barrier and participate in its protective function. They also mediate the processes of signal modulation within the extracellular matrix (ECM). Specific gene variants of collagen polymorphisms participate in the clinical manifestation of the severity of AD and may become candidates to act as biomarkers in the future if more studies support this thesis. Finally, new preparations like nanoliposomes, which contain hydrolyzed collagen, show promise for use as topical emollients. Studies suggest that these could potentially improve the management of local symptoms in AD. A better understanding of collagen-related mechanisms in this disease may lead to the development of more effective therapies and ultimately improve the quality of life for patients suffering from AD.

## References

[1] Nutten S. (2015). Atopic dermatitis: Global epidemiology and risk factors. Ann. Nutr. Metab..

[2] Oliveira A.D.T., Sodré C.S., Ferreira D.C., Abad E.D., Saintive S., Ribeiro M., Cavalcante F.S., Piciani B., Gonçalves L.S. (2018). Oral Aspects Identified in Atopic Dermatitis Patients: A Literature Review. Open Dent. J..

[3] Boguniewicz M., Leung D.Y.M. (2018). Atopic dermatitis: A disease of altered skin barrier and immune dysregulation. Immunol. Rev..

[4] Hulshof L., Overbeek S.A., Wyllie A.L., Chu M.L.J.N., Bogaert D., de Jager W., Knippels L.M.J., Sanders E.A.M., van Aalderen W.M.C., Garssen J. (2018). Exploring Immune Development in Infants with Moderate to Severe Atopic Dermatitis. Front. Immunol..

[5] Murota H., Yamaga K., Ono E., Katayama I. (2018). Sweat in the pathogenesis of atopic dermatitis. Allergol. Int..

[6] Weidinger S., Illig T., Baurecht H., Irvine A.D., Rodriguez E., Diaz-Lacava A., Klopp N., Wagenpfeil S., Zhao Y., Liao H. (2006). Loss-of-function variations within the filaggrin gene predispose for atopic dermatitis with allergic sensitizations. J. Allergy Clin. Immunol..

[7] Macheleidt O., Sandhoff K., Kaiser H.W. (2002). Deficiency of epidermal protein-bound omega-hydroxyceramides in atopic dermatitis. J. Ivestig. Dermatol..

[8] Trzeciak M., Sakowicz-Burkiewicz M., Wasserling M., Dobaczewska D., Gleń J., Nowicki R., Pawelczyk T. (2017). Expression of Cornified Envelope Proteins in Skin and Its Relationship with Atopic Dermatitis Phenotype. Acta Derm Venereol..

[9] Wollenberg A., Bieber T. (2009). Proactive therapy of atopic dermatitis—An emerging concept. Allergy.

[10] Simpson E.L., Hanifin J.M. (2006). Atopic dermatitis. Med. Clin. N. Am..

[11] Agrawal R., Woodfolk J.A. (2014). Skin Barrier Defects in Atopic Dermatitis. Curr. Allergy Asthma Rep..

[12] Guttman-Yassky E., Waldman A. (2017). Atopic Dermatitis: Pathogenesis. Semin. Cutan. Med. Surg..

[13] Brunner P.M., Guttman-Yassky E., Leung D.Y.M. (2017). The immunology of atopic dermatitis and its reversibility with broad-spectrum and targeted therapies. J. Allergy Clin. Immunol..

[14] Czarnowicki T., He H., Krueger J.G., Guttman-Yassky E. (2019). Atopic dermatitis endotypes and implications for targeted therapeutics. J. Allergy Clin. Immunol..

[15] Witte E., Kokolakis G. (2014). IL-19 is a component of the pathogenetic IL-23/IL-17 cascade in psoriasis. J. Investig. Dermatol..

[16] Li K., Seok J., Park K.Y., Yoon Y., Kim K.H., Seo S.J. (2016). Copy-number variation of the filaggrin gene in Korean patients with atopic dermatitis: What really matters, ‘number’ or ‘variation’?. Br. J. Dermatol..

[17] Liu PZhao Y. (2016). Clinical features of adult/adolescent atopic dermatitis and Chinese criteria for atopic dermatitis. Chin. Med. J..

[18] Chan T.C., Sanyal R.D. (2018). Variable T(H)2/T(H)17-skewing places Chinese atopic dermatitis and psoriasis on an inflammatory spectrum. J. Investig. Dermatol..

[19] Kong H.H., Segre J.A. (2012). Skin microbiome: Looking back to move forward. J. Investig. Dermatol..

[20] Kim J.E., Kim H.S. (2019). Microbiome of the Skin and Gut in Atopic Dermatitis (AD): Understanding the Pathophysiology and Finding Novel Management Strategies. J. Clin. Med..

[21] Gilani S.J.K., Gonzalez M., Hussain I., Finlay A., Patel G.K. (2005). *Staphylococcus aureus* re-colonization in atopic dermatitis: Beyond the skin. Clin. Exp. Dermatol..

[22] Paller A.S., Kong H.H., Seed P., Naik S., Scharschmidt T.C., Gallo R.L., Luger T., Irvine A.D. (2019). The Microbiome in Patients with Atopic Dermatitis. J. Allergy Clin. Immunol..

[23] Alexander H., Paller A., Traidl-Hoffmann C., Beck L., De Benedetto A., Dhar S., Girolomoni G., Irvine A., Spuls P., Su J. (2019). The role of bacterial skin infections in atopic dermatitis: Expert statement and review from the International Eczema Council Skin Infection Group. Br. J. Dermatol..

[24] Arseni L., Lombardi A., Orioli D. (2018). From Structure to Phenotype: Impact of Collagen Alterations on Human Health. Int. J. Mol. Sci..

[25] Gunzer M., Friedl P., Niggemann B., Bröcker E.B., Kämpgen E., Zänker K.S. (2000). Migration of dendritic cells within 3-D collagen lattices is dependent on tissue origin, state of maturation, and matrix structure and is maintained by proinflammatory cytokines. J. Leukoc. Biol..

[26] Kisling A., Lust R.M. (2019). What is the role of peptide fragments of collagen I and IV in health and disease?. Life Sci..

[27] Vorstandlechner V., Laggner M., Kalinina P., Haslik W., Radtke C., Shaw L., Lichtenberger B.M., Tschachler E., Ankersmit H.J., Mildner M. (2020). Deciphering the functional heterogeneity of skin fibroblasts using single-cell RNA sequencing. FASEB J..

[28] Bhattacharjee O., Ayyangar U., Kurbet A.S., Ashok D., Raghavan S. (2019). Unraveling the ECM-Immune Cell Crosstalk in Skin Diseases. Front. Cell Devel. Biol..

[29] Meyer M., Müller A.K., Yang J., Ŝulcová J., Werner S. (2011). The role of chronic inflammation in cutaneous fibrosis: Fibroblast growth factor receptor deficiency in keratinocytes as an example. J. Investig. Dermatol. Symp. Proc..

[30] Van Doren S.R. (2015). Matrix metalloproteinase interactions with collagen and elastin. Matrix Biol..

[31] Fidler A.L. (2018). The triple helix of collagens—An ancient protein structure that enabled animal multicellularity and tissue evolution. J. Cell Sci..

[32] Mouw J.K. (2014). Extracellular matrix assembly: A multiscale deconstruction. Nat. Rev. Mol. Cell Biol..

[33] Nielsen S.H. (2023). A fragment of type VI collagen alpha-6 chain is elevated in serum from patients with atopic dermatitis, psoriasis, hidradenitis suppurativa, systemic lupus erythematosus and melanoma. Sci. Rep..

[34] Uitto J. (1989). Connective Tissue Biochemistry of the Aging Dermis: Age-Associated Alterations in Collagen and Elastin. Clin. Geriatr. Med..

[35] Lee J., Jang A., Seo S.J., Myung S.C. (2020). Epigenetic regulation of filaggrin gene expression in human epidermal keratinocytes. Ann. Dermatol..

[36] Nyström A. (2021). Transmembrane collagens-Unexplored mediators of epidermal-dermal communication and tissue homeostasis. Exp. Dermatol..

[37] Harrison I.P. (2018). Hydrogels for Atopic Dermatitis and Wound Management: A Superior Drug Delivery Vehicle. Pharmaceutics.

[38] Suarez-Farinas M., Dhingra N. (2013). Intrinsic atopic dermatitis shows similar TH2 and higher TH17 immune activation compared with extrinsic atopic dermatitis. J. Allergy Clin. Immunol..

[39] Czarnowicki T., Gonzalez J. (2015). Severe atopic dermatitis is characterized by selective expansion of circulating TH2/TC2 and TH22/TC22, but not TH17/TC17, cells within the skin-homing T-cell population. J. Allergy Clin. Immunol..

[40] Pfisterer K. (2021). The Extracellular Matrix in Skin Inflammation and Infection. Front. Cell Dev. Biol..

[41] Szalus K. (2023). The Associations of Single Nucleotide Polymorphisms of the COL3A1, COL6A5, and COL8A1 Genes with Atopic Dermatitis. J. Pers. Med..

[42] Huang F., Wachi S. (2008). Potentiation of IL-19 expression in airway epithelia by IL-17A and IL-4/IL-13: Important implications in asthma. J. Allergy Clin. Immunol..

[43] Sabatelli P., Gara S.K., Grumati P., Urciuolo A., Gualandi F., Curci R., Squarzoni S., Zamparelli A., Martoni E., Merlini L. (2011). Expression of the collagen VI α5 and α6 chains in normal human skin and in skin of patients with collagen VI-related myopathies. J. Investig. Dermatol..

[44] Jung H.J. (2022). The Role of Collagen VI α6 Chain Gene in Atopic Dermatitis. Ann. Dermatol..

[45] Lee Y.I. (2021). Proteoglycan Combined with Hyaluronic Acid and Hydrolyzed Collagen Restores the Skin Barrier in Mild Atopic Dermatitis and Dry, Eczema-Prone Skin: A Pilot Study. Int. J. Mol. Sci..

[46] Makmatov-Rys M. (2023). The Effect of single nucleotide polymorphism in COL23A1 gene on increased HSV-1 susceptibility of human macrophages. Acta Derm. Venereol..

[47] Hakuta A. (2017). Anti-inflammatory effect of collagen tripeptide in atopic dermatitis. J. Dermatol. Sci..

[48] Chopra S. (2023). Identification and characterization of a novel possible genetic risk factor for Eczema Herpeticum. Acta Derm. Venereol..

